# Registration-based transfer listing system and high-tech SME innovation: Research on China’s specialized and sophisticated SMEs listed on the National Equities Exchange and Quotations.

**DOI:** 10.1371/journal.pone.0324208

**Published:** 2025-05-27

**Authors:** Junjie Du, Dong Lu

**Affiliations:** 1 School of Accounting, Southwestern University of Finance and Economics, Chengdu, Sichuan, China; 2 School of Accounting, Southwestern University of Finance and Economics, Chengdu, Sichuan, China; University of Almeria: Universidad de Almeria, SPAIN

## Abstract

In China, high-tech small and medium-sized enterprises (SMEs) are a crucial component of the innovation ecosystem, and how to effectively promote innovation within these firms has become a pressing challenge for the government. Against the backdrop of the registration-based reform, we use a sample of specialized and sophisticated SMEs listed on the National Equities Exchange and Quotations (NEEQ) from 2016 to 2022 to empirically examine the impact of the registration-based transfer listing system (RTLS) on the innovation activities of NEEQ high-tech SMEs. We find that RTLS can enhance innovation input by improving stock liquidity. Meanwhile, we find that RTLS can also promote substantive innovation output by strengthening information disclosure. We further document that the positive effect is more pronounced in highly marketized regions and among non-state-owned enterprises. Further analysis shows that RTLS can enhance innovation efficiency and lessen reliance on fiscal subsidies to promote innovation for NEEQ high-tech SMEs.

## 1. Introduction

Innovation is the key driver of high-quality economic development [1]. In China, high-tech small and medium-sized enterprises (SMEs) play a pivotal role in economic innovation due to their adaptability and flexibility [2,3]. However, these firms face severe debt and equity financing constraints that hinder their innovative growth [4]. Debt financing is often limited for high-tech SMEs due to their small size and higher risk profile, and even when available, given the high capital needs, long cycles, and risks associated with innovation activities [5], creditors tend to restrict its use for innovation. Equity financing is similarly challenging [6], as many high-tech SMEs fall short of the size and profitability requirements for public listing and must instead rely on the National Equities Exchange and Quotations (NEEQ) system. The smaller scale, lower profitability, and less stringent disclosure standards of NEEQ-listed firms lead to its reputation as a "junk firm recycling station," reducing investor interest and stock liquidity, and undermining financing efficiency. Therefore, the dual pressures of difficulties in both debt and equity financing have compelled high-tech SMEs to rely more heavily on government subsidies,

Over the past few years, the government has provided continuous fiscal subsidies to support the innovation activities of high-tech SMEs and alleviate their financing constraints. However, due to the high informational costs between the government and the market [7] and the distortions in market mechanisms caused by government intervention [8], the innovation incentive effect of fiscal subsidies faces dual constraints in terms of ex-ante targeting and ex-post supervision. Specifically, subsidies often fail to be accurately allocated to genuinely innovative firms, and recipient firms frequently fall short of achieving their expected innovation outcomes [9]. This misallocation of resources ultimately distorts resource allocation. Meanwhile, substantial fiscal subsidies impose significant fiscal pressure on the government.

Strengthening the capital market’s support for SME innovation is a critical priority for the Chinese government. The key to addressing the current innovation challenges faced by high-tech SMEs lies in boosting the stock liquidity of NEEQ high-tech SMEs and improving information disclosure quality of these firms. Based on this, the Chinese government officially implemented the registration-based transfer listing system (RTLS) on June 3, 2020. Following several rounds of reforms and improvements, a direct connection mechanism was established between the NEEQ Innovation Layer and the Beijing Stock Exchange (BSE). Specifically, firms in the NEEQ Innovation Layer that meet the BSE listing standards can apply for a direct transfer, bypassing the traditional, complex review processes. Additionally, RTLS imposes stricter information disclosure requirements, compelling firms to disclose essential details, including financial data, innovation project progress, and risk factors, allowing investors to assess the true value and innovation potential of these firms more accurately. Thus, we focus on whether RTLS can enhance the stock liquidity, alleviate financing constraints of NEEQ high-tech SMEs, and thereby promote their innovation input. Meanwhile, we also examine whether RTLS can improve information disclosure standards, increase resource allocation efficiency, and thereby promote substantial innovation output for NEEQ high-tech SMEs.

To investigate the above issues, we use China’s specialized and sophisticated SMEs (characterized by specialization, refinement, distinctiveness, and innovation) listed on the NEEQ from 2016 to 2022 as the research sample, and employ a difference-in-differences (DID) model to examine the impact of RTLS on innovation input and substantial innovation output of NEEQ high-tech SMEs. We find that RTLS can increase innovation input of NEEQ hi-tech SMEs by an average of 0.4% for the treatment group. And we also find that RTLS can increase substantive innovation output of NEEQ hi-tech SMEs by an average of 8.8% for the treatment group. The above conclusions satisfy the parallel trends test and the placebo test. Subsequently, we conduct a series of robustness checks, including substituting the measurement methods of the dependent variables, performing entropy balancing, and excluding the interference of other policies, further confirming the robustness of the baseline results.

Based on the main regression results, we conduct mechanism tests. The mechanism results show that, on the one hand, RTLS can enhance the stock liquidity of NEEQ high-tech SMEs, facilitating their access to innovation funding and thereby increasing their innovation input. On the other hand, RTLS can improve the quality of information disclosure among NEEQ high-tech SMEs, enhancing resource allocation efficiency and ultimately boosting their substantive innovation output.

Furthermore, considering that varying firm characteristics and market environments may exert different influences on the main conclusions of this study, we conduct a heterogeneity analysis by dividing the main sample based on ownership type and the level of marketization. We find that the positive effects on innovation input and substantive innovation output are more pronounced in regions with higher marketization and among non-state-owned enterprises (non-SOEs).

Finally, we further examine the impact of RTLS on the innovation efficiency and dependence on fiscal subsidies to promote innovation of NEEQ high-tech SMEs. Our findings indicate that RTLS can enhance the innovation efficiency of NEEQ high-tech SMEs while reducing their reliance on fiscal subsidies to promote innovation.

Compared to existing literature, this paper offers three contributions. First, our study contributes to the existing literature on registration-based reform. Prior research has primarily focused on its impact on IPO pricing [10], capital market price discovery efficiency[11,12], stock return volatility [13], the cost of equity capital [14], and ESG greenwashing [15]. However, limited attention has been given to the effects of registration-based reform on SMEs. We address this gap by examining the impact of RTLS on the innovation activities of SMEs. By doing so, our research extends the literature on registration-based reform and provides new insights into its implications for SMEs.

Second, our research contributes to the literature on SME innovation. Prior literature has primarily examined the impact of traditional “government-led” policies on SME innovation, including government subsidy policies [16], tax incentives [17], and intellectual property protection policies [18]. However, few studies explore the effect of capital market institutional reforms on SME innovation. By adopting a capital market institutional innovation perspective, this study unveils the mechanism through which RTLS influences SME innovation, contributing to the literature by enriching the understanding of SME innovation drivers from the perspective of “market-driven” policies.

Finally, the findings of this study hold practical significance. While existing literature has thoroughly debated the effectiveness of government subsidies in corporate innovation [19–21], some research suggests that excessive government intervention can significantly hinder SME innovation [22]. From the perspective of capital market reform, we highlight the positive role of “market-driven” policies in fostering SME innovation. This provides policymakers with empirical insights and valuable policy references.

The remainder of this paper is organized as follows. Section 2 introduces the institutional background. Section 3 outlines the hypothesis development. Section 4 presents the research design. Section 5 reports the empirical results. Section 6 concludes the study by summarizing the findings and providing corresponding policy recommendations.

## 2. Institutional background

The NEEQ, BSE, and RTLS collectively form a progressive system within China’s multi-tiered capital market that serves SMEs. As an over-the-counter market, the NEEQ is a crucial component of China’s capital market, designed to facilitate financing for SMEs, particularly high-tech SMEs. The NEEQ is divided into two tiers: the Basic Tier and the Innovation Tier. The Basic Tier has relatively low entry requirements and primarily targets early-stage firms, whereas the Innovation Tier imposes stricter admission criteria, focusing on firms with higher growth potential. However, compared to main board-listed firms, NEEQ-listed firms are generally smaller in scale, exhibit innovation capabilities but face unstable profitability, and demonstrate varying levels of information disclosure quality. Consequently, the NEEQ has long struggled with issues such as low valuation, insufficient stock liquidity, and high financing costs, and has even been derisively referred to by some investors as a “dumping ground for underperforming firms.”

The BSE serves as a key segment of China’s capital market dedicated to facilitating the listing and financing of high-tech SMEs. Unlike the NEEQ, the BSE is part of China’s A-share market. It aims to deepen the reform of the NEEQ and enhance the multi-tiered capital market system. The BSE primarily accommodates high-quality firms from the NEEQ Innovation Tier that meet the eligibility criteria for board transition, providing high-tech SMEs with more efficient financing channels.

**Fig 1:**
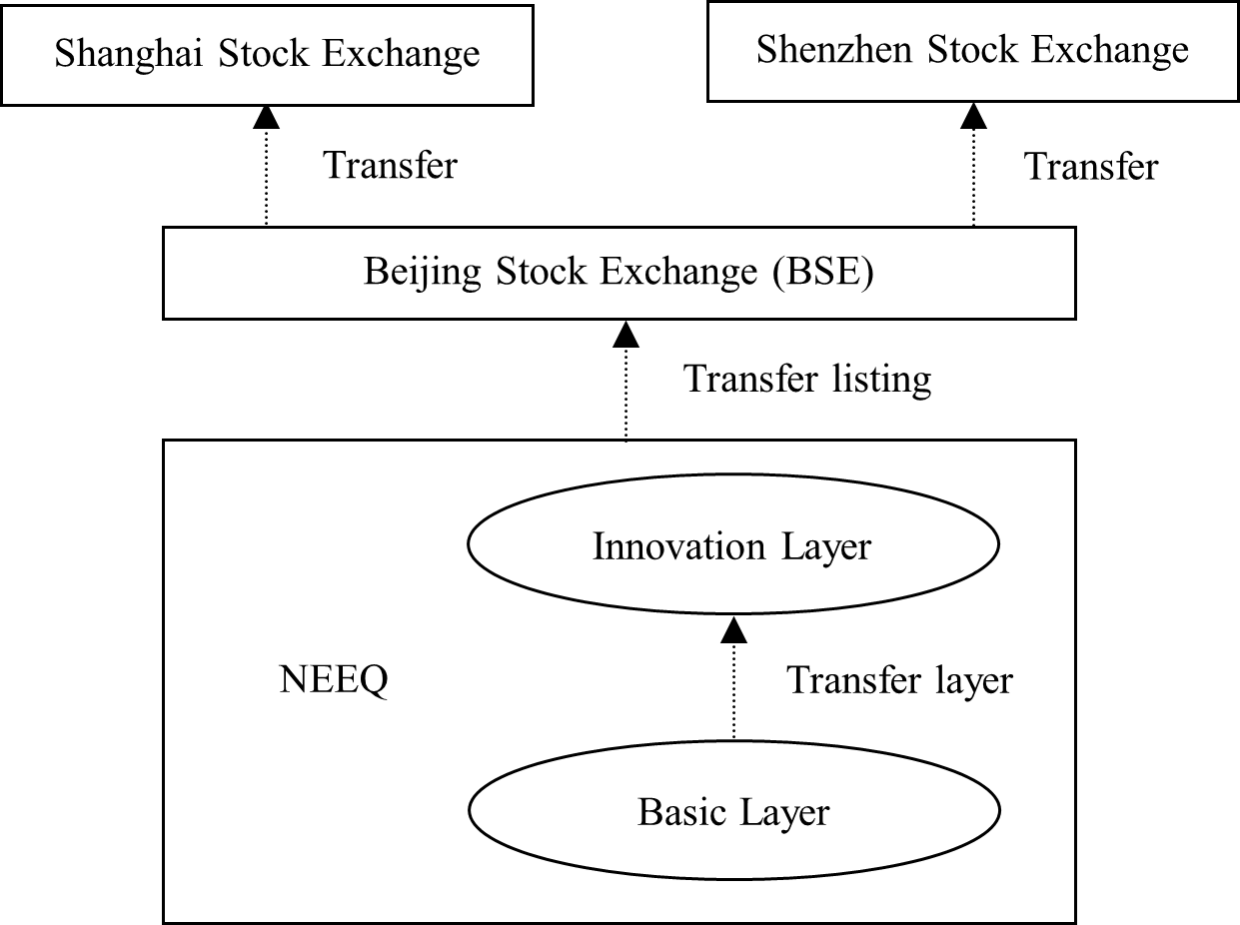
Transfer listing schematic diagram of NEEQ-listed SMEs.

RTLS, introduced on June 3, 2020, serves as a crucial mechanism linking the NEEQ Innovation Tier with the BSE. Prior to its implementation, firms listed on the NEEQ seeking to transition to public markets were required to first delist and undergo a complex, costly, and time-consuming IPO process, posing significant challenges to achieving listed status. Through ongoing reforms, RTLS has established a direct connectivity mechanism between the NEEQ Innovation Tier and the BSE. The transfer listing process for NEEQ-listed SMEs is illustrated in Fig 1. Specifically, firms listed on the NEEQ Innovation Tier for at least one year and meeting the listing requirements for the target board can apply for listing on the BSE, with additional criteria established to assess the innovation capabilities of high-tech SMEs. Furthermore, firms that successfully list on the BSE may subsequently apply to transfer to the Shanghai or Shenzhen Stock Exchange if they meet the respective listing requirements, creating a multi-tiered, progressive pathway for listing. RTLS effectively expands financing opportunities for NEEQ high-tech SMEs, enhancing their access to capital markets.

Simultaneously, RTLS introduces specific regulations on information disclosure to mitigate information asymmetry between investors and NEEQ-listed firms and enhance resource allocation efficiency. On the one hand, RTLS mandates that firms applying for transfer listing ensure their information disclosure is truthful, accurate, and complete, and that it is relevant and comprehensible for significant investor decisions. Stock exchanges conduct multiple rounds of inquiries to verify the authenticity, accuracy, and completeness of disclosures, closely monitoring any instances of false reporting. On the other hand, RTLS assigns increased accountability to intermediary institutions, requiring sponsors and their representatives, accounting firms, law firms, and other securities service providers to oversee and ensure the quality of firms’ information disclosures.

## 3. Hypothesis

### 3.1 Based on market financing

Large firms typically rely on internal resources to support their R&D activities [23]. In contrast, SMEs, due to limited internal funding, tend to utilize established stock markets as a primary source of innovation financing [24–26]. Stock liquidity is the key indicators of capital market quality [27]. The introduction of RTLS can help address the stock liquidity challenges faced by NEEQ high-tech SMEs.

First, RTLS establishes a direct pathway for NEEQ high-tech SMEs to list on main exchanges. The criteria for transfer listing have shifted from a profit-focused approach to an emphasis on innovation and growth potential, with additional innovation requirements specifically tailored for NEEQ high-tech SMEs, thereby increasing the likelihood of listing for them.

**Fig 2:**
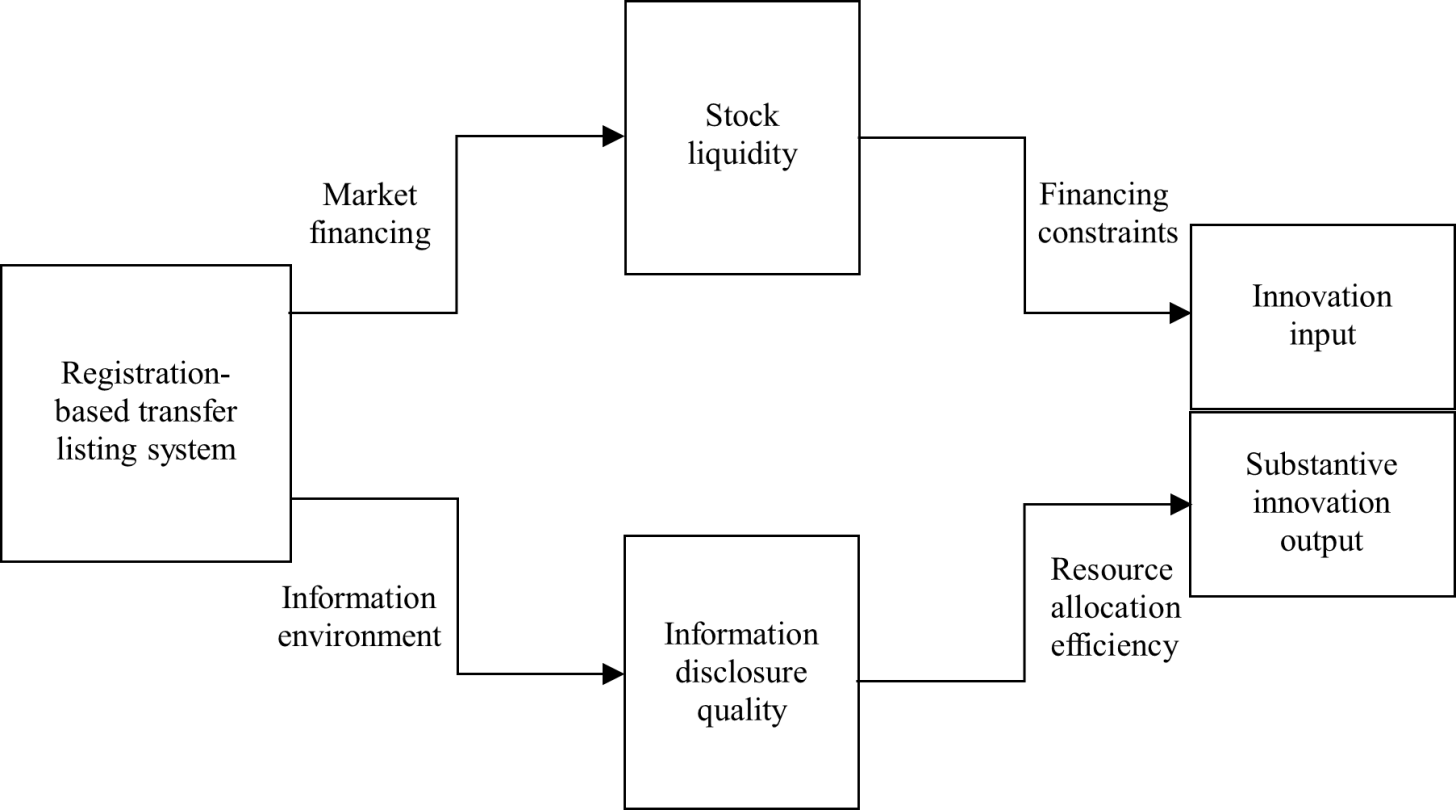
Theoretical framework.

Second, RTLS simplifies the listing process for NEEQ high-tech SMEs. By delegating review authority to the relevant exchanges and replacing substantive reviews with procedural ones, regulatory focus shifts to information disclosure. According to Wind Financial Terminal database, before the registration-based system was implemented, the average time from the initial IPO application to approval for NEEQ firms was 474 days; this period has since shortened to 227 days post-implementation. Thus, RTLS offers listing convenience to NEEQ high-tech SMEs, potentially raising their overall valuation and attracting more external investors. This, in turn, enhances stock liquidity, alleviating financing constraints and fostering innovation input among these firms. The specific theoretical framework is depicted in Fig 2. Based on the above analysis, we propose Hypothesis 1.

**Hypothesis 1.** RTLS can promote innovation input in NEEQ high-tech SMEs.

### 3.2 Based on information environment

Financial markets not only support economic activities by alleviating financing difficulties but also reduce information acquisition costs, enabling more effective project evaluation, selection, and monitoring. This, in turn, enhances resource allocation efficiency [28], which can significantly boost firms’ substantial innovation output [29]. Previously, the NEEQ’s information disclosure framework lacked standardization, resulting in high information opacity and elevated investment risks for investors [30]. Consequently, the price discovery function of the NEEQ was impaired, leading to significant resource misallocation. The information disclosure requirements under RTLS aim to improve the quality of information disclosure for NEEQ high-tech SMEs, thereby mitigating information asymmetry.

First, a key prerequisite for NEEQ high-tech SMEs to transition to listed status is the absence of false disclosures. Additionally, intermediaries effectively monitor these firms’ disclosure practices, increasing the cost of non-compliance. Second, the heightened investor attention and trading activity generated by RTLS provides external oversight for the NEEQ. Given the high entry threshold for NEEQ investors, most are institutional investors, who—according to effective monitoring theory—possess greater expertise in gathering, interpreting, and supervising information, leading to more effective corporate oversight. These institutional investors follow the “Wall Street Rule," influencing corporate governance through frequent trading, or “voting with their feet” [31]. Improved corporate governance reinforces information disclosure practices, thereby enhancing the disclosure quality of NEEQ high-tech SMEs. Improving the information environment optimizes the investment decisions and behaviors of all stakeholders [32], reducing information asymmetry between NEEQ high-tech SMEs and investors. This ultimately increases the efficiency of market-based resource allocation, making it easier for truly innovative firms to access necessary resources, thus promoting substantial innovation output in NEEQ high-tech SMEs. The specific theoretical framework is depicted in Fig 2. Based on the above analysis, we propose Hypothesis 2.

**Hypothesis 2.** RTLS can promote substantial innovation output in NEEQ high-tech SMEs.

## 4. Data and research design

### 4.1 Data source and sample selection

Considering that the innovation capabilities of NEEQ-listed firms vary significantly, with Specialized and Sophisticated SMEs placing greater emphasis on innovation, we choose the above firms as the subjects of our study. To exclude the impact of the 2016 NEEQ tiered system, we select the sample period from 2016 to 2022. The research sample is processed as follows: (1) excluding firms in the financial industry; (2) excluding samples with missing key indicators; (3) excluding ST firms; and (4) performing a two-sided 1% winsorization on all continuous variables. Ultimately, we obtain 3,659 observations for this study. Patent data is manually collected from Tianyancha website; government subsidy data is manually collected from the notes in annual reports; and other data is sourced from the Wind Financial Terminal and China Stock Market & Accounting Research (CSMAR) database.

### 4.2 Model

In selecting the treatment and control groups, we consider the current two-tier structure of the NEEQ, which includes the Basic Tier and the Innovation Tier. Compared to firms in the Basic Tier, firms in the Innovation Tier generally exhibit higher levels of corporate governance and innovation capabilities, making them the primary targets of the RTLS. Furthermore, the latest transfer listing policy stipulates that only firms that have been continuously listed on the Innovation Tier for at least one year are eligible to transfer to the BSE. Therefore, we designate the Innovation Tier as the treatment group and the Basic Tier as the control group. We design the model (1) to test Hypothesis 1 and model (2) to test Hypothesis 2.


$$RD_{it+1}=\omega_{1}+\theta_{1}DID_{it}+\lambda_{1}Controls_{it}+\sum Firm+\sum Year+\varepsilon_{it}$$ (1)

$$Lninno_{it+1}=\omega_{2}+\theta_{2}DID_{it}+\lambda_{2}Controls_{it}+\eta_{i}+\mu_{t}+\varepsilon_{it}$$ (2)

#### 4.3 Definition of variables.

In model (1), the dependent variable is *RD*_*it+1*_, defined as R&D expenditure in period *t + 1* divided by total assets in period *t + 1*. The independent variable is *DID*_*it*_, which equals *Treat*_*i*_×*Post*_*t*_. *Treat*_*i*_ is 1 if the firm belongs to the NEEQ Innovation Tier, and 0 otherwise. *Post*_*t*_ is 1 for years 2020 and onwards, and 0 otherwise. *θ*_*1*_ measures the impact of RTLS on the innovation input of NEEQ high-tech SMEs. It serves as a focal point of this study. According to the theoretical analysis, if RTLS enhances the innovation input of NEEQ high-tech SMEs, we expect *θ*_*1*_ to be significantly positive. We also control for other variables (*Controls*_*it*_) that may affect corporate innovation input, including firm size (*Size*_*it*_), debt-to-asset ratio (*Lev*_*it*_), firm age (*Age*_*it*_), largest shareholder ownership (*Top1*_*it*_), market power (*Market*_*it*_), operating revenue (*Sales*_*it*_), per capita GDP (*LnGDP*_*it*_), return on equity (*ROE*_*it*_), board size (*BOD_Size*_*it*_), and profitability (*Return*_*it*_).

In model (2), the dependent variable is *Lninno*_*it+1*_, defined as the natural logarithm of the number of invention patent applications plus one. *θ*_*2*_ measures the impact of RTLS on the substantive innovation output of NEEQ high-tech SMEs. According to the theoretical analysis, if RTLS enhances the substantive innovation output of NEEQ high-tech SMEs, we expect *θ*_*2*_ to be significantly positive. All other variables are defined as in Model (1). The specific definitions of the variables are provided in Table 1.

**Table 1:** Definition of primary variables.

Variable	Definition
RD	The proportion of R&D expenditure to total assets
Lninno	The natural logarithm of the number of invention patent applications plus one
DID	If firm *i* is in the treatment group (*Treat* = 1) and the year is 2020 or later (*Post* = 1), then *DID* is equals to 1, and 0 otherwise
Size	The natural logarithm of total assets plus one
Lev	Total liabilities divided by total assets
Age	The logarithm of the number of years since the establishment of the firm, plus one
Top1	The proportion of shares held by the largest shareholder
Market	The logarithm of the ratio of operating revenue to operating cost, plus one
Sales	The natural logarithm of the firm’s total operating revenue
LnGDP	The natural logarithm of the per capita GDP of the city where the firm is located plus one
ROE	Net profit divided by average net assets
BOD_Size	The natural logarithm of the number of board members plus one
Return	If the firm’s net profit for the year is greater than 0, it equals 1; otherwise, it equals 0

## 5. Empirical results

### 5.1 Summary statistics

Table 2 presents the results of the descriptive statistics in this study. The mean value of *RD* is 0.051, indicating that the average annual innovation input of firms accounts for approximately 5.1% of total assets during the sample period. The mean value of the original *Lninno* is 4.184, with a standard deviation of 7.996 (original values of invention patents before taking the logarithm), suggesting a significant variation in the number of invention patent applications across firms. The mean value of *DID* is 0.167, indicating that, on average, 16.7% of the total sample was affected by RTLS during the sample period.

**Table 2:** Descriptive statistical analysis.

Variables	Obs	Mean	SD	Min	Median	Max
RD	3659	0.051	0.036	0.005	0.042	0.224
Lninno	3659	1.133	0.914	0.000	1.099	3.178
DID	3659	0.167	0.373	0.000	0.000	1.000
Size	3659	1.184	0.493	0.292	1.118	2.632
Lev	3659	0.422	0.162	0.089	0.424	0.786
Age	3659	2.657	0.377	1.609	2.708	3.367
Top1	3659	0.473	0.176	0.159	0.459	0.906
Market	3659	0.432	0.280	-1.688	0.372	2.390
Sales	3659	0.946	0.439	0.228	0.860	2.345
LnGDP	3659	11.446	0.464	9.900	11.491	12.436
ROE	3659	0.096	0.094	-0.254	0.095	0.370
BOD_Size	3659	1.895	0.161	1.792	1.792	2.303
Return	3659	0.916	0.278	0.000	1.000	1.000

### 5.2 Baseline results

Table 3 presents the main regression results. In column (1), the *DID* coefficient is 0.004, which is significantly positive at the 1% level. This indicates that RTLS can enhance innovation input of NEEQ high-tech SMEs, supporting Hypothesis 1. From an economic perspective, RTLS can lead to an average increase of 0.4% in innovation input for NEEQ high-tech SMEs in the treatment group compared to those in the control group. In column (2), The coefficient of the *DID* is 0.088, which is significantly positive. The result indicates that RTLS can enhance substantive innovation output of NEEQ high-tech SMEs, supporting Hypothesis 2. From an economic perspective, RTLS can lead to an average increase of 8.8% in substantive innovation output for NEEQ high-tech SMEs in the treatment group compared to those in the control group.

**Table 3:** Baseline regression.

	RD_it + 1_	Lninno _it + 1_
	(1)	(2)
DID	0.004***	0.088*
	(0.001)	(0.044)
Size	-0.028***	0.461***
	(0.006)	(0.173)
Lev	-0.009*	-0.122
	(0.005)	(0.257)
Age	-0.002	-0.225
	(0.011)	(0.198)
Top1	0.016	-0.173
	(0.010)	(0.337)
Market	0.006	0.267
	(0.005)	(0.165)
Sales	0.004	0.086
	(0.003)	(0.113)
LnGDP	0.005	-0.067
	(0.004)	(0.074)
ROE	-0.013*	-0.433*
	(0.007)	(0.229)
BOD_Size	0.003	-0.179
	(0.003)	(0.187)
Return	0.001	0.058
	(0.002)	(0.061)
_cons	0.019	2.238*
	(0.037)	(1.322)
Firm	Yes	Yes
Year	Yes	Yes
N	3035	3019
Adj. R^2^	0.810	0.418

Notes: Values in parentheses represent robust standard errors. *, **, and *** denote significance at the 10%, 5%, and 1% levels, respectively.

### 5.3 Parallel trend test

The parallel trends assumption is a prerequisite for the use of the DID model, which means that, prior to the implementation of RTLS, the innovation input trends of the treatment and control groups should satisfy the parallel trends assumption. Therefore, this study follows the approach of Jacobson et al. (1993) [33] and employs an event study methodology to test whether the main regression model in this paper satisfies the parallel trends assumption. The method can be expressed as follows.


$$RD_{it+1}/Lninno_{it+1}=\omega_{3}+\sum {\mathrm{\backslash nolimits}}_{t=-4}^{1}\delta_{t}DID_{it}+\lambda_{3}Controls_{it}+\eta_{i}+\mu_{t}+\varepsilon_{it}$$ (3)

This study takes the first period before RTLS implementation as the baseline. As shown in Table 4, columns (1) and (2) present the results of the parallel trends test for Hypothesis 1 and Hypothesis 2, respectively. Prior to the implementation of RTLS, the *DID* coefficients are not statistically significant. However, after RTLS implementation, the *DID* coefficients are significantly positive, indicating the positive impact of RTLS on the innovation input and substantive innovation output of NEEQ high-tech SMEs. These results suggest that the DID model used in this study satisfies the parallel trends assumption.

**Table 4:** Parallel trend test.

	RD_it + 1_	Lninno _it + 1_
	(1)	(2)
Before4	-0.001	-0.026
	(0.004)	(0.099)
Before3	-0.001	-0.053
	(0.002)	(0.059)
Before2	0.001	0.047
	(0.002)	(0.066)
Current	0.003**	0.080
	(0.002)	(0.063)
After1	0.004***	0.091*
	(0.001)	(0.052)
Size	-0.028***	0.463***
	(0.006)	(0.170)
Lev	-0.009**	-0.131
	(0.004)	(0.264)
Age	-0.002	-0.230
	(0.011)	(0.199)
Top1	0.015	-0.174
	(0.010)	(0.340)
Market	0.006	0.265
	(0.005)	(0.167)
Sales	0.004	0.088
	(0.003)	(0.113)
LnGDP	0.005	-0.067
	(0.004)	(0.073)
ROE	-0.013*	-0.420*
	(0.006)	(0.226)
BOD_Size	0.003	-0.187
	(0.003)	(0.189)
Return	0.001	0.058
	(0.002)	(0.061)
_cons	0.019	2.262*
	(0.038)	(1.346)
Firm	Yes	Yes
Year	Yes	Yes
N	3035	3019
Adj. R^2^	0.809	0.417

Notes: Values in parentheses represent robust standard errors. *, **, and *** denote significance at the 10%, 5%, and 1% levels, respectively.

### 5.4 Placebo tests

**Fig 3:**
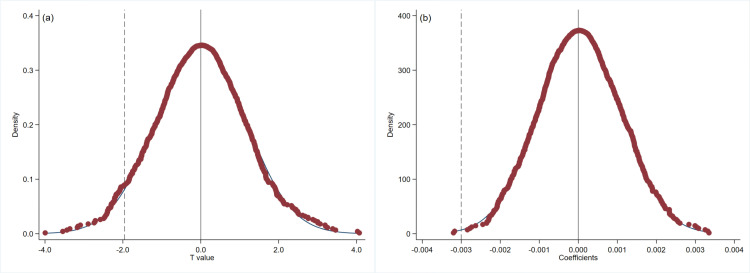
Placebo test results of innovation input. Notes: The placebo test results of the T-value (a) and estimator (b).

**Fig 4:**
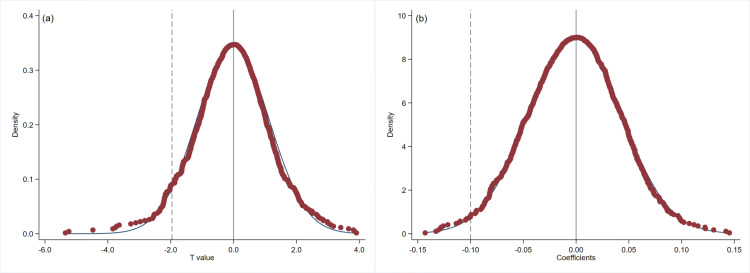
Placebo test results of substantive innovation output. Notes: The placebo test results of the T-value (a) and estimator (b).

To prevent the influence of unobservable variables on the results, we conduct a placebo test. Following the method of Liu and Lu (2015) [34], Cai et al. (2016) [35] and Lu et al. (2022) [36], we randomly select *DID* from the full sample to perform the placebo test on the incentive effect of innovation input and substantive innovation output in NEEQ high-tech SMEs. Fig 3 shows the results of 1000 random samplings for Hypothesis 1 and Fig 4 presents the results of 1000 random samplings for Hypothesis 2. Panel A and Panel B show the results of the t-statistics and the *DID* estimation coefficients, respectively. In the placebo test, the dummy variables *DID* are randomly assigned, and thus, the interaction term is not expected to exert a significant effect on the dependent variable. Consequently, the regression coefficient of the interaction term in the placebo test should cluster around zero in the kernel density plot. Meeting this condition ensures that the model avoids identification bias stemming from omitted variables. In Figs 3 and 4, Both the t-statistics and *DID* estimation coefficients do not significantly deviate from zero, indicating that the placebo test is passed.

### 5.5 Robustness tests

#### 5.5.1 Replacing dependent variables.

First, we replace the dependent variable in Hypothesis 1. Given that the main regression has measured innovation input (*RD*_*it+1*_) using the ratio of R&D investment to total assets, we replace innovation input (*RD*_*it+1*_) with the ratio of R&D investment to operating income in the robustness check, denoted as *Rd*_*it+1*_. Subsequently, we re-estimate Model (1). As shown in Column (1) of Table 5, the *DID* estimate is significantly positive, supporting Hypothesis 1.

**Table 5:** Robustness test results replacing variables and method.

	Replacing dependent variables		Entropy balance test	
	Rd_it + 1_	IR_it + 1_	RD_it + 1_	Lninno _it + 1_
	(1)	(2)	(3)	(4)
DID	0.008**	0.058*	0.002**	0.105**
	(0.003)	(0.033)	(0.001)	(0.049)
Size	0.039***	0.163***	-0.018***	0.762***
	(0.013)	(0.055)	(0.007)	(0.257)
Lev	-0.086***	0.057	-0.009	-0.662**
	(0.021)	(0.077)	(0.011)	(0.316)
Age	-0.021	-0.063	0.003	-0.336
	(0.029)	(0.112)	(0.005)	(0.365)
Top1	0.030**	0.055	0.024***	0.088
	(0.014)	(0.110)	(0.008)	(0.523)
Market	0.019	0.046	0.010*	0.089
	(0.019)	(0.080)	(0.006)	(0.210)
Sales	-0.029***	0.047	-0.001	-0.025
	(0.010)	(0.048)	(0.004)	(0.133)
LnGDP	-0.003	-0.092	0.002	0.118
	(0.004)	(0.058)	(0.004)	(0.166)
ROE	0.012	-0.140	-0.003	-0.155
	(0.020)	(0.112)	(0.007)	(0.230)
BOD_Size	-0.002	-0.102	-0.003	-0.319
	(0.007)	(0.111)	(0.002)	(0.230)
Return	0.005	0.032	-0.000	-0.011
	(0.004)	(0.036)	(0.002)	(0.105)
_cons	0.159*	1.491*	0.042	0.534
	(0.082)	(0.825)	(0.055)	(2.246)
Firm	Yes	Yes	Yes	Yes
Year	Yes	Yes	Yes	Yes
N	3019	3019	3054	3038
Adj. R^2^	0.656	0.278	0.837	0.509

Notes: Values in parentheses represent robust standard errors. *, **, and *** denote significance at the 10%, 5%, and 1% levels, respectively.

Next, we replace the dependent variable in Hypothesis 2. In the previous analysis, the logarithm of patent applications is used as a proxy for substantive innovation output (*Lninno*_*it+1*_). To reflect the proportion of substantive innovation output more comprehensively among all innovation outputs, we use the ratio of the number of invention patent applications in the subsequent period to the total number of patent applications (*IR*_*it+1*_) as the measure of substantive innovation output, and re-estimates Model (2). As shown in Column (2) of Table 5, the *DID* estimate is significantly positive, supporting Hypothesis 2. The above results demonstrate that even after substituting the measurement methods for the dependent variables, the *DID* coefficients still yield significantly positive results, confirming that RTLS for the NEEQ facilitates the innovation input and substantive innovation output of high-tech SMEs listed on the NEEQ.

#### 5.5.2 Entropy balance test.

To reduce the impact of sample selection bias on the research results, this paper adopts entropy balancing, following the method outlined by Hainmueller (2012) [37]. First, we identify the characteristic variables that may introduce bias, and then seek a set of weights to ensure that the experimental and control groups are similar across all characteristics. Next, we assign higher weights to control group samples that are more like the experimental group and estimate the model using weighted regression. The regression results are presented in Columns (3) - (4) of Table 5. The *DID* coefficients are significantly positive, confirming Hypothesis 1 and Hypothesis 2.

#### 5.5.3 Excluding the interference of other policies.

During the sample period, the increase in innovation input and substantive innovation output among NEEQ high-tech SMEs may be attributed to national policies supporting innovation and development of SMEs. To rule out the potential impact of these policies on our conclusions, we collect policy documents issued during the sample period, including the “Law of the People’s Republic of China on Promoting Small and Medium-sized Enterprises (revised in 2017),” the “State Council Opinions on Promoting High-Quality Development of Innovation and Entrepreneurship and Upgrading the ‘Mass Entrepreneurship and Innovation’ Initiative (Guofa [2018] No. 32),” and the “Guiding Opinions on Promoting the Healthy Development of Small and Medium-sized Enterprises” (issued in 2019). Accordingly, we conduct robustness tests on Models (1) and (2) using three subsample periods: 2017–2022, 2018–2022, and 2019–2022. The regression results, as shown in Table 6, indicate that the *DID* coefficient is significantly positive, suggesting that RTLS facilitates innovation input and substantive innovation output among high-tech SMEs listed on the NEEQ.

**Table 6:** Robustness test results excluding the interference of other policies.

	2017-2022		2018-2022		2019-2022	
	RD_it + 1_	Lninno _it + 1_	RD_it + 1_	Lninno _it + 1_	RD_it + 1_	Lninno _it + 1_
	(1)	(2)	(3)	(4)	(5)	(6)
DID	0.003**	0.071*	0.003**	0.084*	0.004***	0.119**
	(0.001)	(0.040)	(0.001)	(0.042)	(0.001)	(0.044)
Size	-0.029***	0.448**	-0.027**	0.585***	-0.006	0.509**
	(0.008)	(0.204)	(0.013)	(0.189)	(0.010)	(0.239)
Lev	-0.004	-0.367	-0.002	-0.596*	-0.006	-0.581*
	(0.005)	(0.294)	(0.007)	(0.315)	(0.008)	(0.309)
Age	-0.015*	-0.338	-0.028***	-0.157	-0.028***	0.282
	(0.008)	(0.272)	(0.005)	(0.318)	(0.010)	(0.520)
Top1	0.010	-0.190	0.017	-0.029	0.030**	0.156
	(0.011)	(0.399)	(0.016)	(0.516)	(0.014)	(0.767)
Market	0.007	0.137	0.008	0.219	0.013*	0.361*
	(0.006)	(0.193)	(0.006)	(0.211)	(0.007)	(0.207)
Sales	0.003	0.113	0.004	0.171	-0.007	0.234
	(0.004)	(0.135)	(0.007)	(0.153)	(0.004)	(0.164)
LnGDP	0.007	-0.086	0.010**	-0.145	0.006***	-0.133
	(0.004)	(0.070)	(0.005)	(0.093)	(0.002)	(0.233)
ROE	-0.007	-0.590**	-0.004	-0.244	0.004	-0.050
	(0.006)	(0.290)	(0.008)	(0.193)	(0.004)	(0.222)
BOD_Size	0.003	-0.295	0.003	-0.268	0.005	-0.556
	(0.004)	(0.177)	(0.005)	(0.281)	(0.007)	(0.342)
Return	-0.000	0.105	-0.001	0.022	-0.001	-0.017
	(0.002)	(0.069)	(0.002)	(0.049)	(0.003)	(0.053)
_cons	0.033	3.102***	0.026	3.062**	0.046*	2.139
	(0.039)	(0.935)	(0.037)	(1.377)	(0.023)	(3.361)
Firm	Yes	Yes	Yes	Yes	Yes	Yes
Year	Yes	Yes	Yes	Yes	Yes	Yes
N	2638	2624	2145	2135	1618	1610
Adj. R^2^	0.816	0.425	0.822	0.451	0.854	0.478

Notes: Values in parentheses represent robust standard errors. *, **, and *** denote significance at the 10%, 5%, and 1% levels, respectively.

### 5.6 Possible mechanisms

We propose that RTLS can promote innovation input of NEEQ high-tech SMEs by improving liquidity on NEEQ and alleviating financing constraints. Besides, RTLS can enhance those firms’ substantive innovation output by improving the quality of information disclosure. This section will test these two mechanisms.

#### 5.6.1 Based on market financing.

Existing literature suggests that stock liquidity can reduce firms’ financing costs, thereby increasing their innovation input [38,39]. RTLS can enhance the stock liquidity of NEEQ high-tech SMEs. To test this mechanism, we examine stock liquidity indicators for those enterprises after the implementation of RTLS, including the Amihud illiquidity measure (*Amihud*), the number of nonzero trading days (*Nonzero*), trading volume (*Volume*), and turnover rate (*TO*). A higher *Amihud* value indicates poorer stock liquidity, while greater *Nonzero*, *Volume*, and *TO* values indicate stronger stock liquidity. Table 7, columns (1) - (4), present the regression results, indicating that RTLS improves the stock liquidity of NEEQ high-tech SMEs.

**Table 7:** Mechanism test results.

	Market financing				Information environment	
	Amihud_it_	Nonzero_it_	Volume_it_	TO_it_	REM_it_	OEO_it_
	(1)	(2)	(3)	(4)	(5)	(6)
DID	-0.019***	0.602***	1.071***	0.055***	-0.022*	-0.302**
	(0.006)	(0.105)	(0.128)	(0.016)	(0.012)	(0.141)
Size	-0.007	0.794***	0.131	-0.079**	0.193***	0.421
	(0.007)	(0.195)	(0.343)	(0.032)	(0.024)	(0.469)
Lev	0.040***	-0.677**	-0.514	0.047	0.122*	-4.739***
	(0.014)	(0.261)	(0.789)	(0.066)	(0.062)	(0.622)
Age	0.021	1.196**	0.718	0.026	-0.018	-1.022
	(0.026)	(0.454)	(1.035)	(0.051)	(0.056)	(1.023)
Top1	-0.033	-2.009**	-1.874	0.097	-0.099	0.346
	(0.029)	(0.877)	(1.391)	(0.108)	(0.071)	(0.597)
Market	-0.011	-0.189	-0.448	-0.019	0.011	1.216***
	(0.009)	(0.153)	(0.280)	(0.018)	(0.036)	(0.444)
Sales	-0.001	0.169	0.593**	0.064***	-0.093***	1.497***
	(0.006)	(0.144)	(0.267)	(0.020)	(0.023)	(0.367)
LnGDP	-0.011	0.184*	0.051	-0.024	-0.011	-0.070
	(0.009)	(0.098)	(0.249)	(0.017)	(0.017)	(0.134)
ROE	-0.000	1.618***	1.370***	0.123**	0.044	-0.716
	(0.025)	(0.327)	(0.507)	(0.053)	(0.061)	(0.695)
BOD_Size	0.018	-0.164	-0.123	-0.007	-0.026	0.358
	(0.011)	(0.199)	(0.422)	(0.036)	(0.019)	(0.337)
Return	0.002	-0.070	0.182	-0.011	0.008	-0.078
	(0.004)	(0.091)	(0.229)	(0.013)	(0.016)	(0.151)
_cons	0.073	-1.968	10.183**	0.272	0.041	8.066***
	(0.089)	(1.554)	(4.019)	(0.333)	(0.143)	(2.924)
Firm	Yes	Yes	Yes	Yes	Yes	Yes
Year	Yes	Yes	Yes	Yes	Yes	Yes
N	2110	2111	2111	2182	3418	2844
Adj. R^2^	0.186	0.652	0.550	0.337	0.315	0.239

Notes: Values in parentheses represent robust standard errors. *, **, and *** denote significance at the 10%, 5%, and 1% levels, respectively.

#### 5.6.2 Based on information environment.

Existing literature confirms that improvements in information disclosure quality help prompt substantive innovation activities [32]. RTLS can enhance firms’ information disclosure quality. To test this mechanism, we examine the information disclosure quality of NEEQ high-tech SMEs after the implementation of RTLS, using real earnings management (*REM*) and earnings opacity (*OEO*) as measures. Higher values of these indicators demonstrate lower information disclosure quality. Table 7, columns (5) - (6), present the regression results, showing that RTLS improves the information disclosure quality of NEEQ high-tech SMEs.

### 5.7 Heterogeneity analysis

Considering that firms with different ownership structures and levels of marketization may face varying financing costs and information disclosure quality [40–42], this section explores how RTLS affects innovation investment and substantive innovation output of NEEQ high-tech SMEs under different ownership structures and marketization levels.

#### 5.7.1 The impact of property rights nature.

Under the context of market-oriented reforms, state-owned enterprises (SOEs) continue to have access to abundant resources. Significant differences exist between SOEs and non-SOEs in terms of financing constraints and innovation-driven dynamics. Consequently, the impact of RTLS on firms’ innovation input and substantive innovation output varies significantly between the two.

First, scarce resources are predominantly controlled by the government, and local government performance objectives lead to greater support for SOEs [43]. As a result, limited resources are disproportionately allocated to SOEs [40], while non-SOEs face higher levels of credit discrimination [44,45]. Thus, non-SOEs experience more severe financing constraints, and improved stock liquidity under RTLS is more likely to enhance their innovation input. Second, compared to SOEs, non-SOEs are subject to fewer social responsibility pressures, such as addressing employment issues and driving local economic growth. Consequently, the improvement in resource allocation efficiency is more likely to promote substantive innovation outcomes in non-SOEs and conduct subsamples test.

To examine the differential effects of RTLS on innovation input and substantive innovation output between SOEs and non-SOEs, this study categorizes the entire sample based on whether the ultimate controller is state capital. We conduct subsample regressions for Models (1) and (2) separately for SOEs and non-SOEs. Columns (1) - (4) of Table 8 present the effects of RTLS on innovation input and substantive innovation output for SOEs and non-SOEs. The p-value of the inter-group coefficient difference test is significantly positive and the *DID* coefficient is only significantly positive for non-SOEs, indicating that RTLS promotes innovation input and substantive innovation output in non-SOEs.

**Table 8:** Heterogeneity test results.

	Property rights nature				Marketization level			
	RD_it + 1_		Lninno _it + 1_		RD _it + 1_		Lninno _it + 1_	
	SOEs	Non-SOEs	SOEs	Non-SOEs	High	Low	High	Low
	(1)	(2)	(3)	(4)	(5)	(6)	(7)	(8)
DID	0.008	0.004***	0.027	0.093**	0.005***	0.000	0.091*	0.048
	(0.006)	(0.001)	(0.244)	(0.045)	(0.001)	(0.002)	(0.047)	(0.116)
Size	-0.015	-0.029***	0.306	0.423**	-0.029***	-0.029***	0.376**	0.637**
	(0.010)	(0.006)	(0.594)	(0.182)	(0.006)	(0.010)	(0.171)	(0.315)
Lev	-0.001	-0.010**	-0.954	-0.105	-0.017***	0.025**	0.073	-0.795
	(0.019)	(0.005)	(1.335)	(0.261)	(0.005)	(0.011)	(0.301)	(0.577)
Age	0.031**	-0.003	-2.857***	-0.113	-0.003	0.002	-0.396*	0.762
	(0.013)	(0.011)	(0.528)	(0.224)	(0.010)	(0.016)	(0.198)	(0.887)
Top1	0.021	0.017*	-1.291	-0.124	0.028**	-0.050***	-0.185	-0.881
	(0.056)	(0.010)	(3.126)	(0.329)	(0.013)	(0.018)	(0.307)	(1.031)
Market	0.003	0.006	-1.894	0.292*	0.014*	-0.005	0.243	0.297
	(0.029)	(0.005)	(1.302)	(0.170)	(0.008)	(0.005)	(0.171)	(0.331)
Sales	-0.007	0.004	-0.197	0.100	0.002	0.006	0.250**	-0.237
	(0.010)	(0.004)	(0.711)	(0.124)	(0.003)	(0.006)	(0.100)	(0.245)
LnGDP	0.009	0.004	1.090**	-0.112	0.008	-0.003	-0.060	0.075
	(0.015)	(0.004)	(0.481)	(0.072)	(0.005)	(0.005)	(0.061)	(0.278)
ROE	-0.004	-0.012*	2.720	-0.485**	-0.019**	0.007	-0.375	-0.687
	(0.033)	(0.007)	(2.557)	(0.234)	(0.008)	(0.012)	(0.229)	(0.649)
BOD_Size	0.024*	0.003	-1.152	-0.157	0.004	0.002	-0.188	0.027
	(0.013)	(0.003)	(1.049)	(0.204)	(0.003)	(0.009)	(0.229)	(0.360)
Return	0.013**	0.001	-0.815	0.074	0.002	-0.001	0.039	0.188
	(0.005)	(0.002)	(0.592)	(0.062)	(0.002)	(0.003)	(0.061)	(0.173)
_cons	-0.186	0.030	0.494	2.387*	-0.024	0.113**	2.558*	-1.914
	(0.181)	(0.040)	(3.048)	(1.302)	(0.049)	(0.055)	(1.322)	(4.131)
Firm	Yes	Yes	Yes	Yes	Yes	Yes	Yes	Yes
Year	Yes	Yes	Yes	Yes	Yes	Yes	Yes	Yes
N	132	2902	132	2886	2369	631	2356	628
Adj. R^2^	0.791	0.810	0.494	0.414	0.818	0.746	0.425	0.386
P value	0.000***		0.051*		0.266		0.398	

Notes: Values in parentheses represent robust standard errors. *, **, and *** denote significance at the 10%, 5%, and 1% levels, respectively.

#### 5.7.2 The impact of marketization level.

Differences in regional resource endowments, policy conditions, and geographic locations result in significant variations in the degree of marketization across regions [46]. Consequently, the impact of RTLS on innovation input and substantive innovation output among NEEQ high-tech SMEs may exhibit heterogeneity depending on the level of marketization in different regions.

First, regions with a high degree of marketization possess more abundant innovation resources. These regions often have well-developed innovation ecosystems, including incubators, accelerators, technology partners, and industrial clusters. Such networks and collaborations facilitate knowledge sharing, resource integration, and mutual innovation promotion. Additionally, highly marketized regions tend to attract a concentration of talented professionals and technological resources, creating an agglomeration effect. The abundance of innovation resources in these areas further attracts more innovation-driven firms, resulting in a higher density of such firms in highly marketized regions. Second, regions with a high degree of marketization typically place greater emphasis on intellectual property (IP) protection and maintain a more robust and stringent IP protection environment. A strong IP protection system provides an institutional foundation for enhancing the efficiency of R&D investment and its subsequent output, enabling enterprises to better translate R&D inputs into performance outcomes [47].

Based on the above analysis, we argue that RTLS’s role in promoting innovation input and substantive innovation output is more pronounced in highly marketized regions. Accordingly, referring to Gao et al. (2023) [48], we divide the full sample into high-marketization and low-marketization subsamples based on the Fan Gang Marketization Index and conduct subsample regressions. Columns (5) - (8) of Table 8 present the regression results. Although the p-value of the inter-group coefficient difference test is not significant, the *DID* coefficient in the high-marketization group is larger and more significant compared to the low-marketization group. This indicates that the implementation of RTLS can enhance innovation input and substantial innovation output for NEEQ high-tech SMEs in high-marketization regions.

### 5.8 Further analysis

#### 5.8.1 RTLS and innovation efficiency.

R&D activities exhibit strong positive externalities, as firms investing heavily in R&D cannot fully capture the residual benefits generated by such activities [49]. Furthermore, the presence of financing constraints further suppresses the innovation incentives of high-tech SMEs. To address the insufficient innovation drive caused by market failures, the government has introduced a series of industrial support policies, including fiscal subsidies and various tax incentives for qualifying firms. As industrial policies become widespread, existing studies find that these policies essentially represent an incomplete contract. The incompleteness of this contract lies in the fact that neither the government nor firms can fully anticipate future events, and no reliable third party exists to ensure the effective enforcement of the contract [50]. This incompleteness leads to information asymmetry during policy implementation, making it difficult to achieve the intended policy objectives and ultimately reducing innovation efficiency [51]. The fundamental reason behind this phenomenon lies in the inherent disadvantage of the government compared to the market in the aspect of information screening.

RTLS aims to address the innovation challenges of high-tech SMEs by reforming the capital market and leveraging market-oriented mechanisms. For NEEQ high-tech SMEs, RTLS has two major implications. On the one hand, the emphasis on information disclosure standards in RTLS improves the quality of information disclosure, forcing them to reduce opportunistic innovation behaviors and thereby enhancing innovation efficiency. On the other hand, the financing convenience and valuation enhancement brought about by RTLS attract more investors, increase stock liquidity, and lower financing costs for NEEQ high-tech SMEs. This provides alternative channels for obtaining innovation funding, enabling them to spontaneously reduce innovation motivated solely by policy incentives and further improve innovation efficiency. To test the validity of the above analysis, we design the following model:


$$Eff_{it+1}=\omega_{4}+\theta_{4}DID_{it}+\lambda_{4}Controls_{it}+\eta_{i}+\mu_{t}+\varepsilon_{it}$$ (4)

Where *Eff*_*it+1*_ represents the innovation efficiency of firm *i* in year *t + 1*, measured by the ratio of invention patents to R&D expenditure. The definitions of other variables are consistent with those in Model (1). Column (1) of Table 9 shows that the coefficient of *DID* is significantly positive at the 1% level, indicating that the implementation of RTLS can improve the innovation efficiency of NEEQ high-tech SMEs.

**Table 9:** Further analysis results.

	Innovation efficiency	Fiscal subsidies	
	Eff_it + 1_	RD _it + 1_	Lninno _it + 1_
	(1)	(2)	(3)
DID	2.047**	0.015***	0.351*
	(0.817)	(0.004)	(0.187)
Lnsub		0.001**	0.009
		(0.000)	(0.015)
DID×Lnsub		-0.002***	-0.051*
		(0.001)	(0.029)
Size	2.789	-0.029***	0.453**
	(3.009)	(0.006)	(0.171)
Lev	-6.957	-0.009*	-0.128
	(5.965)	(0.005)	(0.255)
Age	-17.789	-0.002	-0.227
	(12.454)	(0.011)	(0.199)
Top1	-7.843	0.017	-0.158
	(11.311)	(0.010)	(0.343)
Market	2.279	0.006	0.272
	(2.533)	(0.005)	(0.165)
Sales	-3.695	0.004	0.090
	(2.321)	(0.003)	(0.112)
LnGDP	-5.240***	0.004	-0.084
	(1.241)	(0.004)	(0.078)
ROE	-5.654	-0.013*	-0.439*
	(5.307)	(0.007)	(0.229)
BOD_Size	-2.014	0.003	-0.184
	(2.419)	(0.003)	(0.192)
Return	0.282	0.001	0.055
	(1.469)	(0.002)	(0.061)
_cons	103.590***	0.024	2.397*
	(30.809)	(0.036)	(1.379)
Firm	Yes	Yes	Yes
Year	Yes	Yes	Yes
N	3018	3035	3019
Adj. R^2^	0.380	0.810	0.418

Notes: Values in parentheses represent robust standard errors. *, **, and *** denote significance at the 10%, 5%, and 1% levels, respectively.

#### 5.8.2 RTLS and fiscal subsidies.

Before the implementation of RTLS, high-tech SMEs faced significant challenges in accessing affordable financing, which served as a primary obstacle to their innovation activities [52]. Although the NEEQ provided a channel for equity financing, its relative independence and high investor thresholds led to significant liquidity issues within the market as the number of listed firms increased. To support SMEs’ innovation activities and alleviate their ex-ante financing constraints, the government continuously increased fiscal subsidies.

Regarding the effectiveness of fiscal subsidies in stimulating innovation, two mainstream perspectives exist. The first is the “effective subsidy” view, which posits that fiscal subsidies incentivize firms to increase R&D investment, thereby promoting innovation [53,54]. The second is the “ineffective subsidy” view, which argues that fiscal subsidies disrupt market competition mechanisms, increase transaction costs [51], and lead to rent-seeking behaviors [55], thereby fostering innovation mimicry. This mimicry encourages firms to adopt a “quantity-over-quality” strategy in innovation [56]. The above literature indicates that while fiscal subsidies can alleviate SMEs’ ex-ante financing constraints and thereby promote innovation from a financing perspective, they may distort market resource allocation mechanisms from an agency perspective. The increase in free cash flow resulting from fiscal subsidies exacerbates agency problems, encouraging firms to engage in fraudulent practices to obtain subsidies, which ultimately undermines the efficiency of fiscal subsidies in promoting innovation.

However, following the implementation of RTLS, the improved liquidity of stocks among NEEQ high-tech SMEs may enhance financing convenience. Whether this reduces their reliance on fiscal subsidies for innovation remains an open question. To explore this issue, we construct the following model:


$$\begin{array}{c} RD_{it+1}/Lninno_{it+1}=\omega_{5}+\theta_{5}DID_{it}+\beta_{5}Lnsub_{it}+\gamma_{5}DID_{it}\times Lnsub_{it}\\ +\lambda_{5}Controls_{it}+\eta_{i}+\mu_{t}+\varepsilon_{it} \end{array}$$ (5)

Where *Lnsub*_*it*_ is calculated as the natural logarithm of the total fiscal subsidies received by the firm *i* in the year *t* plus one. To evaluate whether the implementation of RTLS reduces the reliance of NEEQ high-tech SMEs on fiscal subsidies for innovation, we introduce an interaction term between *DID* and *Lnsub*. The focus is on the sign of the interaction term coefficient *γ*_*5*_. If RTLS reduces the reliance of high-tech SMEs on fiscal subsidies for innovation, we expect *γ*_*5*_ to be significantly negative. The definitions of other variables are consistent with those in the main regression model. Columns (2) - (3) of Table 8 present the regression results of Model (5). The results show that the coefficient of *DID×Lnsub* is significantly negative, indicating that the financing convenience brought by RTLS reduces the reliance of high-tech SMEs on fiscal subsidies for innovation.

## 6. Conclusions and policy implications

Using specialized and sophisticated SMEs listed on the NEEQ from 2016 to 2022 as our research sample, we empirically examine the impact of RTLS on high-tech SMEs’ innovation activities. We find that RTLS can enhance innovation input by increasing stock liquidity and promote substantial innovation output through improving information disclosure quality among NEEQ high-tech SMEs. The conclusions remain robust after robustness tests including replacing dependent variables, entropy balance test, and excluding other relative polices. Heterogeneity analysis shows that the positive effects of RTLS on high-tech SMEs’ innovation activities are more pronounced in non-SOEs and regions with higher marketization. Lastly, we also find that RTLS can enhance innovation efficiency and lessen dependence on fiscal subsidies to promote innovation of NEEQ high-tech SMEs. Based on the above conclusions, we propose the following recommendations.

Firstly, compared to government intervention measures such as fiscal subsidies, the market holds more advantages in promoting innovation. On the one hand, financing through financial markets can provide firms with a continuous stream of innovation capital, alleviating fiscal pressures on the government. This represents a more cost-effective and sustainable way of stimulating innovation compared to fiscal subsidies. On the other hand, the market incurs lower informational costs, improving resource allocation efficiency, while robust market oversight can reduce opportunistic innovation behavior by firms. This helps break the current reliance of firms on fiscal subsidies to sustain innovation. Therefore, in encouraging innovation, the government should focus on adhering to market principles and leveraging market mechanisms to drive innovation rather than directly intervening in the innovation process.

Secondly, the government should further refine the positioning of the NEEQ within the capital market and strengthen its role as an innovation financing platform for early-stage high-tech SMEs. For such firms, significant upfront R&D investments and financing constraints pose substantial barriers to innovation input and output. As the financing platform for early-stage high-tech SMEs, the key to the NEEQ’s role lies in improving internal liquidity and trading activity within the board to ensure that firms have access to sustained equity financing. Enhancing the liquidity and activity of the NEEQ board requires greater participation from external investors, which, in turn, depends on the quality of listed firms within the board. To this end, efforts should be made to build a multi-tiered capital market system, attracting more high-potential innovative firms to enter the NEEQ market. This would ensure a steady pipeline of promising firms for higher-tier boards and incentivize top-performing firms within the NEEQ to transition to higher-level boards.

Finally, regulatory authorities should continuously strengthen penalties for violations of information disclosure to firmly delineate “red lines” against corporate fraud. Under the comprehensive reform of the registration-based system, the market will have greater discretion. To maintain the stable and efficient functioning of market rules, it is imperative to strictly regulate corporate information disclosure practices. Regulatory agencies should clearly define the quality requirements for information disclosure within the NEEQ board, increase the cost of non-compliance, and establish strong connections between fraudulent disclosure behaviors and the interests of companies and relevant individuals. In addition to enhancing monetary penalties, reputational and credit costs should also be emphasized, imposing multi-faceted penalties for violations. This approach would foster a sound information disclosure environment within the board.

This study has several limitations, which also suggest directions for future research. First, the sample is restricted to NEEQ high-tech SMEs, excluding non-NEEQ high-tech SMEs. This limitation may reduce the generalizability of the findings to a broader population of high-tech SMEs. Future research could explore the impact of the RTLS on a wider range of high-tech SMEs to enhance the external validity of the conclusions. Second, the sample period spans 2016 to 2022, covering the early stage of RTLS. However, this timeframe may be insufficient to capture the long-term effects of the policy, particularly the sustained innovation performance of firms after transferring to the main board market. Future research could further examine the long-term impact of RTLS on the innovation activities of high-tech SMEs, as well as the evolution of their innovation performance post-transfer. Third, corporate R&D investment and patent innovation encompass multiple categories, but this study does not distinguish between different types of innovation activities. Future research could conduct a more granular analysis of the heterogeneous effects of RTLS on various types of innovation. Finally, potential unobserved factors (e.g., firms opting for transfer listing may inherently possess stronger innovation capabilities, or the influence of other concurrent policies) could introduce estimation bias in this study. Future research could employ more robust identification strategies to address endogeneity concerns and further validate the causal impact of RTLS on the innovation activities of high-tech SMEs.

## Supporting information

S1 Dataset. (DTA)
